# Transcription factor and microRNA-regulated network motifs for cancer and signal transduction networks

**DOI:** 10.1186/1752-0509-9-S1-S5

**Published:** 2015-01-21

**Authors:** Wen-Tsong Hsieh, Ke-Rung Tzeng, Jin-Shuei Ciou, Jeffrey JP Tsai, Nilubon Kurubanjerdjit, Chien-Hung Huang, Ka-Lok Ng

**Affiliations:** 1 Department of Pharmacology, China Medical University, Taiwan 40402; 2 Department of Biomedical Informatics, Asia University, Taiwan 41354; 3 School of Information Technology, Mae Fah Luang University, Chiang Rai, Thailand 57100; 4 Department of Computer Science and Information Engineering, National Formosa University, Taiwan 632; 5 Department of Medical Research, China Medical University Hospital, China Medical University, Taichung 40402, Taiwan

## Abstract

**Abstract:**

**Background**

Molecular networks are the basis of biological processes. Such networks can be decomposed into smaller modules, also known as network motifs. These motifs show interesting dynamical behaviors, in which co-operativity effects between the motif components play a critical role in human diseases. We have developed a motif-searching algorithm, which is able to identify common motif types from the cancer networks and signal transduction networks (STNs). Some of the network motifs are interconnected which can be merged together and form more complex structures, the so-called coupled motif structures (CMS). These structures exhibit mixed dynamical behavior, which may lead biological organisms to perform specific functions.

**Results**

In this study, we integrate transcription factors (TFs), microRNAs (miRNAs), miRNA targets and network motifs information to build the cancer-related TF-miRNA-motif networks (TMMN). This allows us to examine the role of network motifs in cancer formation at different levels of regulation, i.e. transcription initiation (TF → miRNA), gene-gene interaction (CMS), and post-transcriptional regulation (miRNA → target genes). Among the cancer networks and STNs we considered, it is found that there is a substantial amount of crosstalking through motif interconnections, in particular, the crosstalk between prostate cancer network and PI3K-Akt STN.

**Conclusions:**

To validate the role of network motifs in cancer formation, several examples are presented which demonstrated the effectiveness of the present approach. A web-based platform has been set up which can be accessed at: http://ppi.bioinfo.asia.edu.tw/pathway/. It is very likely that our results can supply very specific CMS missing information for certain cancer types, it is an indispensable tool for cancer biology research.

## Background

Molecular networks are formed by the interaction between biomolecules are the basis of biological processes. These networks include but not limited to protein-protein interaction networks (PPIN), signal transduction networks (STNs), gene regulatory networks (GRN), and metabolic networks (MN). The network consists of a large number of bio-molecules, interacting with each other give rise to biological responses and stabilities. Network components perform their function by cooperating with each other. Such networks can be decomposed into smaller biological modules,also known as network motifs.

Graph theory approach is a powerful tool for investigating the underlying global and local topological structures of molecular networks; such as, analyzing the yeast PPIN [1] and MN [2,3]. Examples of local structures are: auto-regulation loop (ARL, either catalytic or repression), feedback loop (FBL), feed-forward loop (FFL, either coherent or incoherent), bi-fan and single-input motif (SIM) [4-6]. These five network motifs are responsible for a large portion of molecular adjustments when the host is subjected to changes in the external environment (e.g. temperature, chemical concentrations), cell differentiation, development, and signal transduction [7].

Such network motifs are known to have interesting dynamical properties. Besides topological consideration, the dynamical behavior of the motifs can be formulated by a system of ordinary differential equations, where the solutions described certain biological functionalities. For instance, it has been shown that; 1) the FBL is capable of directing bacterial chemotaxis [7], 2) the coherent FFL (cFFL) with 'AND' logic is capable of filtering out transient spikes of input activity [8,9], perform sign sensitive delay [8,10], and 3) the incoherent FFL (iFFL) is capable of accelerating response times [8,11]. Therefore, identifying different network motif types is the first step towards a better understanding of network biology at a system level.

Previous studies have reported certain motifs are commonly found in organisms, such as the FFL is found in *E. coli *[9], in other bacteria [12], in yeasts [13,14] and higher organisms transcriptional regulatory network [15-17]; FBL and FFL also occur in different types of biological networks, such as neural networks and PPIN [18-20]. It is note that there was a work claimed that network motifs do not necessary determine biological functions, there is no characteristic behavior for network motifs [21], while other works [8,22,23] reported opposite results.

Cancer is both a genetic and epigenetic disease. Genetic damage or mutation induced by carcinogens is a possible cause for cancer formation. Monogenic disease traits are rare; it is known that the causes of cancers are polygenic and through gene-gene interaction in general. To get a better understanding of the role of network motifs in cancer biology at a system level, in a 2012 work [24], four motif types, i.e. ARL, FBL, FFL and bi-fan, were identified for six cancer diseases.

Network motifs do not perform biological functions independently, instead motifs are interconnected which lead to observed phenotypic changes. We name these interconnections, the coupled motif structures (CMS). CMS is called motif-motif interaction (MMI) pairs in our previous work [24]. Biological organisms may use coupled motifs to perform specific functions; for instance, coupled FBL form dynamic motifs for cellular networks [25] and shown oscillatory behavior [26].

### Network motifs and signal transduction networks (STNs)

STNs play an essential role in cancer formation. External chemical factor binds to the cell membrane receptor, the chemical signals get transmitted through protein-protein interaction, or post-translation modification, pass on to the transcription factors, imported into the nuclei, which activate or inhibit cancer-related genes. The cause of cancer is due to the malfunction of genetic components of the STNs; such as, Jak-Stat, MAPK, NFkB, PI3K-Akt, Ras, Wnt [27]. Once a component of the STN is affected, the signal would propagate and get amplified; hence, induced anti-apoptosis effect, which leads to cancer eventually.

In this paper, we extended our previous work [24] by identifying five motif types for all the available STNs. During the preparation of the present work, we came across an article written by Chen et al., [28] where the authors have developed a method, called "Selection of Significant Expression-Correlation Differential Motifs" (SSECDM) to study breast cancer. Their work applies a network motif-based approach, and combines STN and high-throughput gene expression data to distinguish breast cancer patients from normal patients.

### Network motifs, microRNAs and transcription factors

In recent years, there is an increasing number of works on examining microRNA-regulated network motifs. Micro-ribonucleic acid (miRNAs) are small, endogenous molecules of ribonucleic acid around 20 to 24 base pairs long that regulate gene expression at a post-transcriptional or translational level [29].

In a recent work by Siciliano et al., [30], the authors have shown that miRNAs confer phenotypic robustness to transcription regulation networks by suppressing fluctuations in protein levels. Also, it has reported that miRNA-mediated FFLs have the effect of bufering the network against phenotypic variation [31]. For instance, hsa-miR-15a involves in cell cycle progression through its interaction with the FFL [32]. There is also a study reported the principles of miRNA regulation in cell STNs [33]. Furthermore, many reports have suggested that aberrant miRNA expression is associated with tumor progression and metastasis. MiRNAs could cause cancers by targeting oncogenes (OCG) or tumor suppressor genes (TSG) [34,35].In another work published in 2013 [36], we have reported the results of miRNA-regulated network motifs for cancer networks obtained from KEGG [37].

Transcription factors (TFs) also play an important role in GRN. In a recent work, the web-based platform named CircuitsDB [38] was released, which provided FFL motif information built from TFs, miRNAs, and genes. In another work [39], the authors constructed a TF-miRNA-gene network (TMG-net) for colorectal and breast cancer by combining experimentally validated and confidently inferring regulatory relations, i.e. miRNA→gene, TF → gene and TF → miRNA interactions.

We propose to build a TF-miRNA-motif networks (TMMN) for cancer diseases. To the best of our knowledge, TMMN is probably the first structure constructed to address the relationships between TFs, miRNAs, CMS, cancer networks and STNs. Furthermore, since cancer networks are highly coupled with the STNs through motif interconnection, we introduce a measure called Jaccard Index (JI) to quantify the degree of crosstalking.

In order to identify network motifs, one needs to collect the regulatory relation between two genetic elements. In the last few years, we began to see many progresses in identifying biological network motifs using network motif prediction tools. However, most of the motifs searching results are based on missing or false negative regulatory relations (see Figure 5 in [40]). If any one of the gene regulatory pair is uncertain, then any motif derived from that is meaningless; therefore, a large collection of highly confident regulatory relations is necessary.

The main advantage of the present computation is that the gene-gene regulatory relations provided by KEGG are experimentally verified, which are highly reliable records. From the biological point of view, these collections of regulatory pairs permit *in silico *researchers to obtain reliable network motif results.

In Section 2, we give a description of the input data and the methods used in this paper. In Section 3, results for cancer-related network motifs, CMS, TMMN, gene set enrichment analysis and several cancer-related motif examples are reported. We conclude in the final section.

## Methods

The cancer networks and STNs information used in this study are downloaded from KEGG (July 2013 version). KEGG integrates genomic, chemical, and systemic functional information to compose a biological database resource.

### Outline of workflow

In the last few years, many biochemical pathways information are released by the KEGG database [37], which are prepared in the XML format. Now, KEGG provides very detail regulatory information among the molecules. For example, KEGG delivers the following information on; 1) PPI (PPrel) including both of the activation and inhibition events, 2) gene expression interactions (GErel) including expression and depression events, 3) post-translational modification (PTM, i.e. PPrel with activation or inhibit phosphorylation), and 4) protein-compound interactions (PCrel with activation or inhibition).

A total of 20 cancer networks and 24 STNs have been processed. Given the regulatory relationships between two genetic components, one can reconstruct network motifs using the graph theory approach. The present study addresses the following issues;

(i) collect highly confident regulatory relations from cancer networks and STNs,

(ii) analyze the abundance of five common types of network motifs,

(iii) merge interconnected motif types to form CMS,

(iv) perform gene set enrichment analysis for CMS,

(v) construct TMMN,

(vi) perform text mining to validate the motif results, and

(vii) quantify crosstalking between cancer networks and STNs.

### Identifying major types of network motifs

There are a number of publicly available network motif detection tools, namely MFINDER [13], MAVISTO [41], FANMOD [42], NetMatch [43], and SNAVI [44]. The main disadvantage of using MFINDER and MAVISTO for network motif detection is that they are comparably slow and scale poorly as the subgraph size increases [22,42]. We have performed a trial study using FANMOD with KEGG data as input, the tool reports subgraphs that occur significantly more often than in random networks. The tool does not provide information on; 1) how many subgraphs are found, and 2) subgraph's nodes identities. In other words, no detail of real motif is supplied. For instance, the output file of FANMOD reports certain motif information, such as frequency of occurrence, Z-value and *p*-value, however, it does not report nodes identities, then one does not know which genetic elements belong to the motif. In other words, given the pairwise information as input, FANMOD can predict over-represented motifs with certain level of accuracy, but it does not report nodes identities.

Also, FANMOD has certain limitation, for instance, it cannot identify motifs with size one and two, i.e. auto-regulation loop and feedback loop. This can be done with the adjacency matrix description. More details are given in the 'Results' section Table 2.

**Table 2:** A comparison of motif finding by the adjacency matrix approach and FANMOD

approach	motif	AML	Glioma	Melanoma	NSCLC	PC	RCC
Adjacency Matrix	FFL	***0***^§^	***0***^§^	***0***^§^	1	1	1
	bi-fan	***1***^§^	***1***^§^	1	1	***0***^§^	***0***^§^

FANMOD	FFL	***0***^§^	***0***^§^	***0***^§^	0 (FN)	0 (FN)	0 (FN)
	
	bi-fan	***1***^§^	***1***^§^	0 (FN)	0 (FN)	***0***^§^	***0***^§^
	Non bi-fan	2(2)*	1(1)*	0(0)*	2(2)*	3(3)*	3(1)*

^§^given a fixed motif size, the italic and underlined fonts denote the results uisng adjacency matrix approach are consistent with those of FANMON, i.e. true positive or true negative events

* the first number denotes the number of identified motifs, the number inside the parenthesis denotes the total number of motif pattern found in the cancer type

Because of this limitation, we have developed a motif searching algorithm, which is able to process KEGG networks, such as; the 'pathways in cancer (overview)' for human, and found a cFFL that involves genes PKC, Ras and Raf. It is interesting to note that this loop participates in coordination of crosstalk between the Ras/Raf and PKC pathways [23,45].

We also tested our motif-searching algorithm for the plant pathogen interaction network, and found two FFLs, where the first FFL involves CNGCs with Ca^++^, CDPK and Rboh, and the second FFL involves MEKK1, MKK1 and MPK4. It is known that the first FFL is associated with Ca^++ ^signaling [46] whereas the second FFL that involves MEKK1, MKK1 and MPK4 is associated with plant immune responses [47-49]. This demonstrates the usefulness of identifying or matching network motifs with functional biological modules.

In the graph theory approach, each bio-molecule is represented as a node and regulatory relation as an edge. One constructs an adjacency matrix to represent the network. In the adjacency matrix a value of one and infinity (for convenient a very large number is used in programming) is assigned to represent direct regulation and non-regulating nodes respectively. For node that is interacting with itself a value of one is assigned. Row and column indices denote the upstream and downstream node respectively. Below we briefly described how to perform the motif search.

#### ARL

This motif type involves a self-regulated gene. Non-zero diagonal elements in the adjacency matrix represent this type of motif. The time complexity is *O*(*n*).

#### FBL

This motif type involves two genes regulate each other. For any location(*i*, *j*) in the adjacency matrix, if the term of (*i*,*j*) is '1' and that of (*j*,*i*) is also '1', then genes *i *and *j *form a FBL. Since there are C(*n*,2) combinations to be tested, the time complexity is *O*(*n*^2^).

#### FFL

This motif type involves three genes regulating each other. Depending on the activation or suppression order, this motif type can be further divided into the so-called cFFL, and iFFL.

For any triple set (*i*,*j*,*k*), if the terms of (*i*,*j*), (*j,i*),(*i*,*k*),(*k*,*i*), (*j*,*k*),(*k*,*j*) are all of '1', then genes *i *, *j *and *k *form a FFL. Since there are C(*n*,3)*6 combinations to be tested, the time complexity is *O*(*n*^3^).

#### Bi-fan

Bi-fan motif denotes a topology where two genes regulate the same other two genes.

Select any two rows in the adjacency matrix which have the value of '1' appear at the same column more than one time. Check whether these two rows are connected, if not, then determine which two columns have the value of '1' in both rows. The time complexity is *O*(*n*^3^). To identify all bi-fan motifs, there are C(n,2)*C(n-2,2) combinations to check, so the time complexity is *O *(*n*^4^).

#### SIM

SIM motif denotes a topology where a master gene regulates multiple downstream genes.

Select any row in the adjacency matrix and count how many '1' appear in the row. Since there are at most *n *'1's in a row and *n *rows to search; therefore, the time complexity is *O*(*n*^2^).

### Coupled motif structures (CMS)

Some of the network motifs are interconnected which lead to observed phenotypic change. The present study identifies possible CMS for cancer networks. As a preliminary study, the following six types of CMS are considered; i.e. FBL-FBL, FFL-FFL, bi-fan bi-fan, FBL-FFL, FBL-bi-fan and FFL-bi-fan. To obtain such structures, gene names of; 1) same motif type, and 2) different motif types, are pairwise compared. Given the CMS, it enables reconstructing the global architecture of the whole network from a bottom-up approach. More complex CMS are also identified, which can be visualized in our web platform.

The following pseudo-code was designed to identify the six types of CMS.

Input: The network A with *n *nodes and all basic network motifs (ARL, FBL, FFL, Bi-fan and SIM) of A.

Output: All CMS of network A

Begin

   For *i *= 1 to n do

      Loop

         If any two network motifs or CMS which include common node *i *could be

         merged to form a meaningful CMS, then merging these two subgraphs to form

         larger CMS;

      Until no more basic network motifs or CMS including node *i *could be merged;

   End of For loop

End

A complex network may have underlying topological structures, which can be characterized by certain topological parameters. We applied the SBEToolbox [50] to compute several topological parameters, i.e. size, maximum degree, bridging centrality (*BRC*) and degree centrality (*DC*), for the CMS.

Size of the network is given by the largest connected cluster. Maximum degree of a node is node with the highest number of connections.

The bridging coefficient of a node *i *is defined by:

(1) $$BCO\left(i\right)=\frac{d{\left(i\right)}^{-1}}{\underset{i\in N\left(i\right)}{\sum }\frac{1}{d\left(i\right)}}$$

where *d*(*i*) is the degree of node *i*, and *N*(*i*) is the set of neighbors of node *i*. Bridging centrality *BRC*(*i*) for node *i *is defined by

BRC(i)=BC(i)×BCO(i)

The betweenness centrality *BC*(*i*) of a node *i *is computed as follows:

(2) $$BC\left(i\right)=\underset{s\neq i\neq t}{\sum }\left(\frac{\sigma_{st}\left(i\right)}{\sigma_{st}}\right)$$

where *s *and *t *are nodes in the network different from *i*, σ_st _denotes the number of shortest paths from *s *to *t*, and σ_st _(*i*) is the number of shortest paths from *s *to *t *that pass through *i*. *BRC *is the average of *BRC(i) *over all *i*.

Degree centrality of a node *i*, *DC*(*i*), denotes the node degree of node *i*. The *DC *of node *i *in a network is defined by:

(3) $$DC\left(i\right)=\frac{\underset{j}{\sum }A_{ij}}{N-1}$$

where *N *denotes the total number of nodes in the network and *A_ij _*is the corresponding entry value in the adjacency matrix *A*. *DC *is the average of *DC(i) *over all *i*.

### MiRNA-regulated network motifs

It is known that miRNA plays a crucial role in controlling gene expression and biological process through its interaction with network motifs. For instance, hsa-miR-15a involves in cell cycle progression [32] through its interaction with the FFL. In particular, we are interested in miRNA target genes that are related to cancer formation, i.e. OCG and TSG.

Most miRNAs show reduced expression during cancer formation; while some are overexpressed in cancers. MiR-155 and its host gene, B-cell integration cluster (BIC), are highly expressed due to MYB regulates BIC in chronic lymphocytic leukemia [51]. Another example is the miR-17-92 cluster, which is activated by the OCG c-Myc and is highly expressed in B-cell lymphoma. Members of the miR-17-92 cluster (miR-19a and miR-19b) are essential to mediate the oncogenic activity of the entire cluster by down-regulated the expression of the TSG, Pten [52]. These studies indicate that some miRNAs may act as OCGs and involve in the initiation and progression of cancers.

Cancer gene data are obtained from the Tumor Associated Gene (TAG) database [53], Memorial Sloan-Kettering Cancer Center (MSKCC) [54] and National Yang Ming University, Taiwan [55]. After removing overlapped information among the three datasets, we have collected a total of 659 OCGs, 1023 TAGs and 151 cancer-related genes. MiRNA target gene information are obtained from miRTarBase (version 4.5) [56] and TarBase (version 5) [57].

To construct TMMN, the TF-regulated miRNA data are retrieved from Chipbase [58]. Since miRNA target genes information are known; then, by matching the cancer motifs or CMS results, we obtained cancer-specific TMMN. In addition, we labeled target genes as OCGs or TSGs if they can be found in our cancer gene set collection.

### Gene set enrichment analysis

Functional annotation of dense PPI module is given by the Database for Annotation, Visualization and Integrated Discovery, i.e., DAVID http://david.abcc.ncifcrf.gov/, which accepts batch annotation and conducts gene set enrichment analysis. Set of CMS involves in a particular cancer network was submitted to DAVID for clustering of the annotation terms and enriched pathways. With such analysis, enriched pathways and biological processes related to the cancer network are obtained.

There are several studies on integrating TF, miRNA and target genes expression profile to construct miRNA-regulated modules for cancer diseases. Zhang et al. [59] applied Sparse Network-regularized Multiple Nonnegative Matrix Factorization (SNMNMF) algorithm to identify miRNA regulatory modules by combining expression profiles of both miRNAs and genes, gene-gene interaction (GGI) and DNA-protein interaction. The study had shown that miRNA-gene modules are enriched in (i) genomics miRNA clusters, (ii) known functional annotations, and (iii) cancer diseases.

Le et al. [60] developed the regression-based model called PIMiM (Protein Interaction-based MicroRNA Modules) to predict miRNA-regulated modules by integrating expression profiles of both miRNAs and genes, sequence-based predictions of miRNA-mRNA interactions and protein-protein interactions data. Using ovarian cancer as a case study, PIMiM had demonstrated that it is able to identify cancer-specific miRNAs, presence of expression coherence between miRNA and mRNA, and enriched functional description.

Li et al. [61] proposed Mirsynergy which applied a two-stage clustering approach to integrate m/miRNA expression profile, target site information and gene-gene interaction (GGI) to infer miRNA regulatory modules (MiRMs).

Our results differ in several aspects, (i) TMMN can provide regulatory order among GGI, (ii) both TF → miRNA and miRNA → gene information are obtained from experimentally verified database, instead of prediction, (iii) we also knew that the target gene is an OCG or TSG; these information are definite not available in those studies [59-61].

### Signal transduction networks (STNs)

Twenty-four STNs are retrieved from KEGG, where only 13 STNs are found to compose of the proposed motif types. To quantify the number of common motif nodes share between cancer networks and STNs, we characterized that using the Jaccard index, *JI*, which is given by:

(4) $$JI\left(A,B\right)=\frac{\left|A\cap B\right|}{\left|A|\cup |B|-|A\cap B\right|}$$

where |*A *∩ *B*|, |*A*| and |*B*| denote the cardinality of *A *∩ *B *, |*A*| and |*B*| respectively. *A *and *B *denote the sets of motif nodes found in a cancer network and a STN respectively.

## Results

### The results of major types of network motifs

A total of 20 cancer networks have been processed, only seven networks; i.e. pathways in cancer, glioma, acute myeloid leukemia (AML), melanoma, renal cell carcinoma (RCC), non-small cell lung cancer (NSCLC), and prostate cancer (PC), have identifiable motifs. Table 1 presents the results of the five motif types for cancer networks and STNs. Our results suggested that the number of bi-fans and SIM motifs outnumber other motif types. Both of ARL and FBL motifs are rare events.

**Table 1:** 

A The total number of the five motif types identified for cancer networks and STNs					
	ARL	FBL	FFL	bi-fan	SIM
**Cancer networks**					

Pathways in cancer	0	0	1	73	27

AML	0	0	0	9	12

Glioma	0	0	0	9	6

Melanoma	0	0	0	1	4

NSCLC	0	1	2	4	7

PC	0	0	1	0	5

RCC	0	0	1	0	3

**Signal transduction networks (STNs)**					

Erbb	0	0	5	69	17

FoxO	0	0	3	0	3

Hippo	0	0	2	0	8

Jak-Stat	0	0	0	4	3

Mapk	0	0	1	6	32

PI3k-Akt	0	0	1	1	10

Rap1	0	0	0	1	13

Ras	0	0	2	15	18

TGF_Beta	0	0	0	1	7

TNF	0	0	0	1	11

TCS	0	0	0	3	35

VEGF	0	0	2	0	5

Wnt	0	0	11	0	7

* TCS denotes the Two-component system					

B The total number of SIM motifs identified in cancer networks and STNs					

**Cancer networks**					SIM

Basal cell carcinoma					1

Bladder cancer					1

Chronic myeloid leukemia					4

Colorectal cancer					4

Endometrial cancer					4

Pancreatic cancer					8

Small cell lung cancer					2

**Signal transduction networks (STNs)**					9

Calcium signaling					3

Hedgehog					6

HIF-1					6

mTOR					10

NFkB					2

Notch					2

Phosphatidylinositol signaling system					

We note that the SIM motif is a more common motif, which is the only identifiable motif type for seven other cancer networks and seven other STNs. In other words, SIM can be found in 14 out of the 20 cancer networks, and 20 out of the 24 STNs. The results are presented in Table 1B.

Our approach, using adjacency matrix, allow us to identify exact motifs, hence, no *p*-values are associated with the findings. In order to compare our results with the randomization approach, we performed motif finding for the six cancer types using FANMOD. Default setting for FANMOD are: *p*-value threshold is 0.05 and number of randomized samples is 1000. We compare the motif finding by our approach and FANMOD, where the results are given in Table 2.

It is evident from the table that FANMOD is not able to identify any FFL motif for NSCLC, PC and RCC, i.e. false negative (FN) events. For motifs with size four, our approach can identify bi-fan structure only, whereas FANMOD can predict more motif patterns. FANMOD predicted bi-fan motif for AML and glioma, which is in line with our findings, i.e. true positive events. FANMOD did not identify any bi-fan motif in PC and RCC, i.e. true negative events, which is in line with our approach.

But, FANMOD found SIM (size of four) for RCC, which is false positive events. Also, it fails to find bi-fan motif for melanoma and NSCLC, i.e. false negative events.

The last row in Table 2 summarized motif patterns with size four identified by FANMOD. These findings indicated that FANMOD performed well in identifying motif pattern with size four except in RCC, i.e. 3(1)* means that among the three motif patterns only one pattern is realized.

Certain network motifs are recorded as cancer-related modules by using the text mining tool, AliBaba http://alibaba.informatik.hu-berlin.de/. AliBaba is a web-based text mining service based on PubMed database, which displays the search result in form of a graph. The following criteria are assumed for literature text mining; 1) for FBL, both nodes are found, 2) for FFL, at least two nodes are found, 3) for bi-fans, at least two nodes are found, and 4) for SIM, since it is a bipartite graph, at least one node in each layer can be found. Table 3 summarized the text mining results, which satisfy the above criteria; for instance, at least 62 publications recorded SIM for the AML disease.

**Table 3:** Cancer-related motifs that are reported in literature

Cancer	ARL	FBL	FFL	Bi-fan	SIM
AML	0	0	0	20	62

Glioma	0	0	0	29	4

Melanoma	0	0	0	2	0

NSCLC	0	3	2	40	37

PC	0	0	2	0	4

RCC	0	0	20	0	22

Our collection of motifs can provide additional details that are not reported in the literature. As a first example, a previous study demonstrated that PI3k/Akt is an important influential factor in cancer, but PDPK1 was not known for its influence in cancer formation [19]. Our study showed that PI3K, Akt3, and PDPK1 form a cFFL; all involving in prostate cancer formation.

As another example, we have identified that PKC and Ras are the upstream regulators of Raf in the MAPK STN, and these three genes form a cFFL. It has reported [62] that Ras-Raf-MAPK is an important pathway in apoptosis suppression. Here we are able to add PKC, which acts as an upstream regulator, is a missing component in the literature.

As a third case, PI3K3CA and PDPK1 (also known as PDK1) are the upstream regulators of Akt, and these three genes form a cFFL in the PI3K-Akt STN. As Fresno et al. [63] stated that PI3K-Akt STN components are frequently altered in human cancers, such as AML, NSCLC, PC and RCC.

### The results of coupled motif structures (CMS)

Table 4 summarizes the results of the six possible types of CMS. The bi-fan bi-fan CMS is the dominant type among all the possibilities. In particular, the Erbb STN has the highest number of bi-fan bi-fan and FFL-bi-fan interconnected structures. This is because the Erbb STN has multiple layers of bi-fan structure, plus bi-fan is the dominant motif type. More complex CMS can be constructed by merging three or more different motif types.

**Table 4:** The results of the six types of CMS for cancer networks and STNs

	FBL-FBL	FFL-FFL	bi-fan-bi-fan	FBL-FFL	FBL-bi-fan	FFL-bi-fan	size	max deg	*BRC*	*DC*
Cancer networks										

AML	0	0	17	0	0	0	22	7	0.310	0.116

Glioma	0	0	36	0	0	0	8	4	0.0443	0.393

Melanoma	0	0	0	0	0	0	4	2	0.0678	0.400

NSCLC	0	1	6	0	4	2	18	6	0.0309	0.150

PC	0	0	0	0	0	0	18	11	0.0137	0.111

RCC	0	0	0	0	0	0	6	5	0.0075	0.333

Median, *α*							13	5.5	0.0376	0.242

Signal transduction networks (STNs)										

Erbb	0	10	1607	0	0	111	31	13	0.0106	0.123

FoxO	0	2	0	0	0	0	34	31	0.00074	0.064

Hippo	0	10	0	0	0	0	12	6	0.0765	0.167

Jak-Stat	0	0	3	0	0	0	16	6	0.0461	0.158

Mapk	0	0	6	0	0	0	72	13	0.0154	0.039

PI3k-Akt	0	0	0	0	0	0	39	15	0.00878	0.058

Rap1	0	0	0	0	0	0	26	15	0.0161	0.080

Ras	0	1	105	0	0	7	39	14	0.0107	0.082

TGF_Beta	0	0	0	0	0	0	5	3	0.0678	0.250

TNF	0	0	0	0	0	0	12	5	0.0379	0.167

TCS	0	0	3	0	0	0	11	8	0.0333	0.182

VEGF	0	1	0	0	0	0	19	8	0.0146	0.123

Wnt	0	24	0	0	0	0	24	9	0.0106	0.123

Median, *β*							32.5	13.5	0.00158	0.081

*γ*							2.50	2.46	0.419	0.335

To address the difference of cancer networks and STNs CMS, we compared the results of their size, maximum degree, *BRC *and *DC*. Let *α *and *β *be the medians of the above four parameters for cancer networks and STNs respectively, and the ratio *γ *is defined by *β*/*α*.

From Table 4 we found that the *γ *values for the size and maximum degree are about 2.5 times bigger for STNs. This implies that STNs CMS incline to form bigger modules and higher gene-gene interactions. However, the ratio for *DC *and *BRC *are 0.335 and 0.419, respectively. The results appear to suggest that cancer networks have higher degree centrality and bridging coefficients. It is known that *DC *shows that an important node is involved in a large number of interactions; whereas, a bridging node is a node connecting densely connected components in a graph. The present analysis revealed that highly interacting nodes and bridging nodes appear to be important components in cancer networks.

### Construction of TF-miRNA-motif networks

The studied cancer network motifs are targeted by multiple miRNAs. Table 5 summarizes the results of these post-transcriptional modification events, i.e. miRNA →motif. In Table 5 the miRNA column represents the total number of miRNAs involve in targeting the motif types. The FBL, FFL and bi-fan columns list the total number of miRNAs involve in regulating those three network motifs respectively. In summary, the miRNA-motif regulatory relations can be classified into three classes, i.e. one-to-many, many-to-one and many-to-many. Certain miRNAs can target multiple motifs (one-to-many), some miRNAs target the same motif (many-to-one), and a few miRNAs can target multiple motifs (many-to-many). In the FBL, FFL, and bi-fan columns, the first and second numbers denote inter-motif and intra-motif regulation respectively. Inter-motif regulation represents the number of miRNAs involve in targeting multiple motifs, whereas intra-motif regulation denotes the number of miRNAs involves in targeting different members of the same motif. For the AML cancer, there are 27 miRNAs involve in regulating multiple bi-fan motifs, and seven miRNAs involve in regulating different targets of the same bi-fan motif.

**Table 5:** Mirna-regulated cancer network motifs

Cancer	miRNA	FBL	FFL	bi-fan
AML	80	0	0	27/7

Glioma	133	0	0	7/0

Melanoma	131	0	0	8/1

NSCLC	92	6/0	1/0	13/1

PC	126	0	1/0	0

RCC	44	0	15/1	0

**Figure 1:**
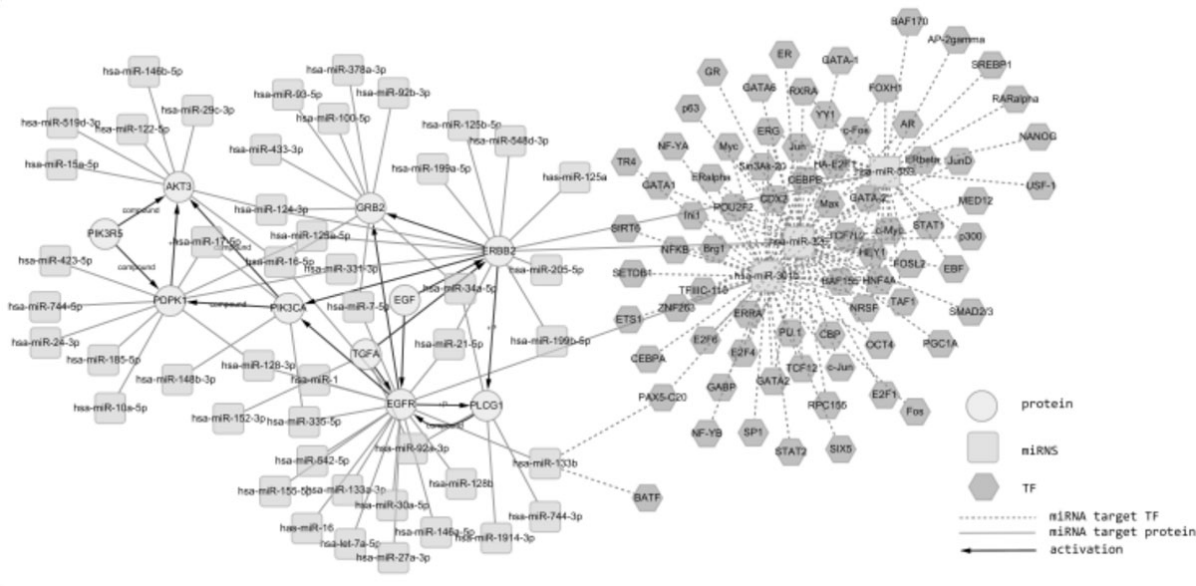
**TMMN for NSCLC network displayed using Cytoscape**. Square node and hexagon denote miRNA and target gene respectively, Circular shape denotes transcription factor. Compound, and p+ represent compound and phosphorylation event respectively. Compound interaction denotes interaction with an intermediate molecule, mostly chemical compound.

By integrating the transcription initiation data, i.e. TF →miRNA events, Figure 1 is a graphical display of TMMN for NSCLC using Cytoscape. Cytoscape http://www.cytoscape.org/ is a useful tool for visualizing molecular interaction network and observing the correlation between molecules.

In order to facilitate the Cytoscape displaying part, we provide two options: (i) low resolution and (ii) high resolution, for the user to view our results. Lower resolution image file allows the user to view TMMN in a faster pace.

As we shown in Table 5 certain bi-fan motifs are highly regulated by miRNAs. Given that a network motif can perform specific biological function, it is suggested that regulating TMMN may result in observable phenotypic effects.

Using the text mining tool, AliBaba, it was found that the Akt expression is significantly correlated with TGFA and EGFR in NSCLC [64]. Our motif searching result indicates that EGF and TGFA are the upstream regulators of EGFR and ERBB2 in NSCLC, in which these four genes form a bi-fan motif. From Figure 1, one can conclude the following pathway, i.e. TGFA → EGFR → PI3K3CA → Akt, which is consistent with Refs. [64,65] description. Our finding not only provides the missing genetic part, PI3KCA; which is not reported in the literature, but also reveals the genetic regulatory order. Again, this illustrates the potential practical application of our results.

### The results of enrichment analysis

Functional annotations of the cancer network motifs are based on gene set enrichment analysis by implementing DAVID. Tables 6 and 7 summarized the gene set enrichment analysis results of the AML and NSCLC networks respectively, with p-value less than or equal to 0.05. Over-represented Gene Ontology [66] biological process (BP), molecular function (MF), cellular component (CC) and KEGG pathway are reported. Because of the limitation of space, the results of gene set enrichment analysis for the other cancer networks are not reported, but it can be accessed in our web-based platform.

**Table 6:** The gene set enrichment analysis results for the AML network motifs

Annotation cluster	Enrichment source	Involving genes	% of the total genes
GO_BP	cellular process	15	93.75%

GO_CC	intracellular	15	93.75%

GO_MF	protein binding	15	93.75%

KEGG	Acute myeloid leukemia	16	100.00%

KEGG	Pathways in cancer	16	100.00%

**Table 7:** The gene set enrichment analysis results for the NSCLC network motifs

Annotation cluster	Enrichment source	Involving genes	% of the total genes
KEGG	NSCLC	10	100.00%

KEGG	ErbB signaling pathway	9	90.00%

KEGG	Glioma	8	80.00%

KEGG	Pathways in cancer	9	90.00%

From Table 7 it is found that giloma is another enriched network in addition to the NSCLC network. This suggested that the same CMS involves in different cancer types, which may hint for disease comorbidity study.

### The results of crosstalk between cancer networks and STNs

Table 8 summarized the *JI *scores for the crosstalk between six cancer networks and 13 STNs, i.e. a total of 78 combinations. The first row and the first column list cancer types and the STNS respectively. Entries in Table 8 represents the JI associated with a STN and the corresponding cancer disease. It is found that most of the entries are non-zero, which indicated that cancer networks are highly coupled with STNs through motif interconnections. For non-zero JI values, the values range from 0.013 to 0.184, where crosstalking between PC and PI3K-Akt has the highest JI value. There are several studies have examined this before; for instance, targeting the PI3K-Akt-mTOR pathway in PC as a clinical treatment [67-69], PI3K pathway is dominant over androgen receptor signaling in PC [70], and activation of PI3K pathway promotes PC cell invasion [71]. The second highest JI belongs to the crosstalk between NSCLC network and ErbB2 STN. Both Erbb and EGFR are mutated in many epithelial tumors; such as, NSCLC and breast cancer [72].

**Table 8:** The Jaccard index for crosstalking of six cancer networks and 13 stns

	AML	Glioma	Melanoma	NSCLC	PC	RCC
Erbb	0.090	0.103	0.096	0.173	0.167	0.109

FoxO	0.024	0	0.023	0.045	0.055	0.021

Hippo	0.026	0	0.025	0.016	0.019	0.022

Jak-Stat	0.082	0.024	0.076	0.083	0.066	0.082

Mapk	0.030	0.027	0.036	0.033	0.050	0.049

PI3k-Akt	0.096	0.046	0.103	0.132	0.184	0.072

Rap1	0.037	0.051	0.034	0.044	0.036	0.032

Ras	0.082	0.038	0.089	0.096	0.088	0.073

TGF_Beta	0	0	0.015	0	0.022	0.027

TNF	0.014	0.020	0.013	0.034	0.019	0.024

TCS	0	0	0	0	0	0

VEGF	0.024	0.154	0.058	0.149	0.053	0.038

Wnt	0.052	0.018	0.049	0.031	0.067	0.056

A web-based interface has been set up for query, and can be accessed at: http://ppi.bioinfo.asia.edu.tw/pathway/. The platform provides useful information according to various cancer types and STNs search. First, for a specific cancer type or STN, user can search for known regulatory relations using the 'Gene-Gene Interaction' button. The platform will return, 1) PPrel, 2) GErel, 3) PTM, and 4) PCrel information. Second, under the 'Cancer regulation motif' or 'Signal transduction network' button, user can select a cancer type or STN, the platform will return all the identified motifs. Third, user can search for TF-regulated miRNA and inter-motif miRNA-regulated gene information from our web platform. Fourth, TMMN can be visualized on-line, which is displayed in Cytoscape format. This information can be adopted to elucidate the role of motifs in cancer formation. Finally, the platform provides PubMed literature ID hyperlinks for the motifs, this allows the users to continue their studies.

## Conclusions

The major conclusions drawn from our results are as follows. First, the bi-fan and SIM motifs are two of the most frequently found motifs in cancer networks and STNs. Second, in the seven cancer networks, the bi-fan bi-fan coupling structure is more probable than the other types. Third, miRNA mediates inter-motif regulation is more often than intra-motif regulation. Fourth, we have examined the role of network motifs in cancer formation at different levels of regulation, i.e. transcription initiation (TF → miRNA), gene-gene interaction (CMS), and post-transcriptional regulation (miRNA → target genes). Fifth, highly interacting nodes and bridging nodes appear to be important components in cancer networks. Sixth, based on the *JI *calculation, there is a substantial amount of crosstalk between cancer networks and the STNs.

By integrating TFs, miRNAs and motif information, cancer-specific TMMN are constructed. Results are deployed as a web-based platform. The platform is unique in the sense that it provides experimentally validated network motif information. Our algorithm can be easily applied to any other networks, once the binary interaction information is available.

As we have indicated in four case studies, it is very likely that our collection of CMS can supply very specific missing information for certain cancer networks; hence, it is an indispensable tool for cancer biology research.

## Competing interests

The authors declare that they have no competing interests.

## Authors' contributions

WTH^* ^and KRT^* ^are the first authors and contributed equally to the work. WTH helped designing the study and provided specific domain knowledge on cancer biology. KRT carried out data retrieval, data analysesandprogram coding. JSC helped performing simulation studies and conducted the enrichment analysis. JJPT oversee the work and proofread the article. NK helped setting up the web site and provide guidence on database management. CHH^§ ^and KLN^§ ^are the corresponding authors of the article, they designed the study, provided insight for all discussions and drafted the manuscript. CHH also providedinstructions on designing the algorithms. All authors read andapproved the manuscript.
